# Partner violence surrounding divorce: A record‐linkage study of wives and their husbands

**DOI:** 10.1111/jomf.12881

**Published:** 2022-09-06

**Authors:** Elina Einiö, Niina Metsä‐Simola, Mikko Aaltonen, Elina Hiltunen, Pekka Martikainen

**Affiliations:** ^1^ Population Research Unit, Faculty of Social Sciences University of Helsinki Helsinki Finland; ^2^ Law School, Faculty of Social Sciences and Business Studies University of Eastern Finland Joensuu Finland

**Keywords:** crime, divorce, domestic violence, intimate partner violence, marital relations, motherhood

## Abstract

**Objective:**

This study analyzes the victimization trajectories of partner violence against women surrounding divorce, depending on whether the couple has children together.

**Background:**

Prior studies have found that partner violence is associated with an increased risk of divorce. No study has assessed the victimization trajectories surrounding divorce for women with and without children, although women with children may remain at higher risk of violence following divorce.

**Method:**

Using Finnish record‐linkage data of 22,468 divorced and 333,542 continuously married women and their husbands, we used repeated‐measures logistic regression analyses to assess changes in victimization for partner violence before and after divorce. The outcomes considered were police‐reported crimes committed by husbands against their wives and hospital‐treated assault injuries recorded for wives.

**Results:**

The risk of crime victimization for partner assault was already elevated from 2 to 3 years before divorce, peaked in the year prior to divorce, and then mainly leveled off 1–2 years after divorce. Hospital data show that the time of the greatest risk was from 6 to 12 months before divorce, when divorce is usually filed for. Women with younger children experienced elevated risks of physical violence shortly before divorce and remained at higher risk of menace than women without children for a year after divorce.

**Conclusion:**

Divorcing women committed assaults against their husbands, but these were mostly accompanied by victimization, suggesting that resistant violence was common for women as perpetrators. Women with a history of victimization need support, especially at the starts of their divorce processes.

## INTRODUCTION

Individuals who have experienced the dissolution of a marriage have poorer health outcomes compared with continuously married individuals (Afifi et al., 2006; Breslau et al., 2011; Grundy & Tomassini, 2010; Joung et al., 1997; Meadows et al., 2008; Metsä‐Simola & Martikainen, 2013; Strohschein et al., 2005). These differences in health result from health changes at the time of divorce, declines in health after the divorce, as well as from a higher likelihood of divorce among those with poorer health (Amato, 2000; Butterworth & Rodgers, 2008; Idstad et al., 2015; Kalmijn, 2017; Metsä‐Simola et al., 2018; Metsä‐Simola & Martikainen, 2013; Wade & Pevalin, 2004). Women are indicated to experience larger material losses following divorce (Garvin et al., 1993; Lillard & Waite, 1995; Pienta et al., 2000), whereas the loss of social support is viewed as more important for the well‐being of divorced men (Gerstel et al., 1985; Lee et al., 2005; Umberson, 1992).

However, less is known about changes in intimate partner violence surrounding the dissolution of a marriage, although partner violence is known to increase the risk of divorce (Bowlus & Seitz, 2006; Kingston‐Riechers, 2001), given that women may leave husbands who act violently. However, partner violence against estranged wives might not stop after marital dissolution (Brownridge, 2006), as indicated by homicide statistics and convenience samples from medical clinics (Hotton, 2001; Johnson & Hotton, 2003; Kershner et al., 1998; Lehti, 2015). It is unclear whether the risk of intimate partner violence among individuals who experience divorce is higher before or after the dissolution of a marriage, and to what extent the risk decreases with time since divorce. Despite the fact that women in intact marriages are shown to face less partner aggression than those who are separated or divorced (Brownridge et al., 2008), these findings, based on different individuals, also reflect unobserved heterogeneity between individuals and their partners. Divorcing individuals might have already had elevated levels of victimization when their marriages were intact but cross‐sectional comparisons do not show it.

Using multiple data sources from Finland—medical records (2007–2017) on female patients and police‐reported data (2011–2017) on victims of intimate partner violence and their perpetrators—the present study assessed how the risk of victimization changes before and after divorce for divorcing women, as compared with continuously married women. We also assessed whether the victimization trajectories are different for women with and without children, given that women with children are more likely than childless women to stay in contact with their estranged husbands (Brownridge, 2006; Fischer et al., 2005). We also aim at studying violent offending of divorcing women and the extent to which it could have arisen in reaction to their husband's use of partner violence. To our knowledge, this is the first population‐based record‐linkage study with repeated outcome measures of victimization for and perpetration of partner violence observed during the transition into divorce, allowing not only examining the time of the greatest risk for women but also their violence against their husbands.

## BACKGROUND

### The Finnish context

Homicide data for Finnish women show that the perpetrator is often a current or former husband in crimes that led to the victim's death. Between 2003 and 2013, approximately 65% of women who were killed in Finland were killed by a current or former husband, a common‐law husband, or a boyfriend (Lehti, 2015). It is the second leading homicide type, preceded only by homicides in which a man kills his male acquaintance or a male friend (Lehti, 2012). In the United States, for example, approximately 45% of women who were killed in 2007 were killed by a current or former spouse or a boyfriend or girlfriend (Catalano et al., 2009). In 2013, the United Nations' Human Rights Committee addressed its concerns to Finland on violence against women and recommended actions to increase the number of shelters for victims of partner violence (United Nations Human Rights Committee, 2011, 2013). Also historically, Finland has been an outlier in the Nordic context (Denmark, Iceland, Norway, and Sweden) because its homicide rate for women has been disproportionately high (Kivivuori & Lehti, 2011), compared with what would have been expected solely on the basis of gender equality. In Finland, nonlethal violence precedes approximately 80% of homicides by intimate partners (National Institute for Health and Welfare, 2021), and it is therefore important to have studies with the aim of identifying how the violence varies surrounding family crises, such as divorce.

### Coercive controlling violence, violent resistance, or situational violence

In his theoretical framework, Johnson (2008) distinguished among different types of partner violence: intimate terrorism, violent resistance, and situational couple violence. The violence was defined by the extent to which the perpetrator and his or her partner used violence motivated by attempts to control the relationship. Intimate terrorism—also known as coercive controlling violence—involves physical violence deployed in the service of general control over one's partner, and the victimized partner does not normally use violence against the perpetrator. Physical violence is described as just one tactic among multiple nonviolent tactics, including emotional abuse, minimizing, denying, blaming, intimidation, isolation, and threats (Johnson, 2008; Nielsen et al., 2016). In violent resistance, the victimized resister's physical violence arises in reaction to the perpetrator's use of coercive controlling violence (Johnson, 2008).

According to Johnson (2008), however, situational couple violence is the most common form of partner violence. The element that distinguishes situational violence from other forms of violence is that it is not motivated by an interest in exerting general control over one's partner in the long run but rather by different motives that arise in reaction to situationally provoked tensions. The violence is situationally provoked when the conflicts or arguments escalate to physical violence once one or both members of the couple react in a violent manner. Although situational couple violence may only be a frustrated expression of anger in order to win an argument or to get the partner's attention, it may also involve intentions to injure the partner in the context of an emotionally tense situation (Johnson, 2008). In a situation of an approaching divorce, the members of the couple might experience heightened levels of stress that cause them to forget their long‐term conciliatory goals, in the sense that short‐term desires to win or alter the situation escalate to violent actions. Previous studies provide support for the idea that divorce is one of the most stressful life processes (Holmes & Rahe, 1967; Noone, 2017; Rahe et al., 2000), and it could provoke irrational behavior. However, it is also possible that one member of the couple rationally desires that the other one does not leave him or her, and is therefore motivated by attempts to regain control over the partner and therefore to ignore criminal and social sanctions that may follow these types of violent actions. For example, Nielsen et al. (2016) have suggested that both coercive controlling violence and situational couple violence can arise or exacerbate in frequency or severity during relationship dissolution.

### Marital status and partner violence

Most cross‐sectional studies of partner violence indicate that marital status and physical partner aggression are related, in the sense that women in intact marriages face less partner aggression than those who are separated or divorced. For example, in a study of women seeking care from medical clinics and food program sites in Minnesota, United States, Kershner et al. (1998) showed that separating women were 6.5 times more likely and divorced women 2.5 times more likely to experience intimate partner violence in the past year, as compared with married women. In a survey of women who were treated at community hospital emergency departments in Pennsylvania and California, Dearwater et al. (1998) found that ending a relationship within the past year was associated with a higher prevalence of intimate partner abuse in the past year. Relatively similarly, in a population‐based survey from Canada, Romans et al. (2007) found that married women and those in common‐law marriage were less likely to experience physical or sexual partner violence, compared with divorced, separated, or unmarried women. Furthermore, in another population‐based cross‐sectional survey—from Sweden—Lövestad and Krantz (2012) indicated that being married or in a stable relationship contributed to decrease the risk of partner assault or sexual coercion. On the contrary, a cross‐cultural study of 19 countries by Bernards and Graham (2013) called into question the common belief that women in stable relationships experience less partner aggression everywhere. They indicated that in India and Uganda divorced or separated women were less likely than married women to report being the victim of partner violence. These unexpected findings were statistically not significant, however. In addition, homicide statistics from Canada, the United States, and Australia consistently indicate that lethal violence following marital disruption is more common among separated than divorced women, indicating that the commencement of a divorce process puts women at risk for partner aggression (Brownridge, 2006; Hotton, 2001).

In addition to previous statistical studies of the association between marital status and partner violence, several risk assessment tools have been developed to assess both an offender's risk of reoffending and a victim's risk of repeat victimization. These tools have been developed, based on empirically and theoretically guided evidence, for various frontline professionals, including social workers, medical doctors, police officers, and criminal justice professionals, to predict and reduce partner assault recidivism or lethality (Graham et al., 2021; Messing & Thaller, 2015; Storey et al., 2014; Turner et al., 2019). In addition to using information on an offender's history of domestic violence, some of the risk assessment tools developed in North America, including the Danger Assessment (DA) tool for lethality and the Domestic Violence Screening Instrument (DVSI) for serious reoffending, include items that identify recent separation from a partner (up to 1 year) as a risk factor for re‐assault (Messing & Thaller, 2015). Furthermore, in a validation study of the United Kingdom's risk assessment tool—Domestic Abuse, Stalking and Honor Based Violence (DASH)—Almond et al. (2017) showed that a victim's attempt to separate or separation predicted recidivism. However, some of the instruments, such as the Ontario Domestic Assault Risk Assessment (ODARA) tool, excluded information on prior separation, given that the item showed poor predictive validity in a real sample of offenders (Hilton et al., 2004).

Even though most cross‐sectional surveys, homicide statistics, and risk assessment tools indicate that divorce can be an especially dangerous time for women, to our knowledge, no prior population‐based study has longitudinally followed for changes in victimization for partner violence over the transition to divorce. It remains unclear whether and to what extent separated or divorced women's risk of victimization is already elevated during their marriages, given that partner violence is known to predict the risk of marital dissolution (Bowlus & Seitz, 2006; Kingston‐Riechers, 2001).

### Children and partner violence

Previous empirical studies have provided mixed results regarding the association between having children in the household and intimate partner violence against women. Some cross‐sectional studies, based on both population‐based samples (Romans et al., 2007; Vest et al., 2002) and convenience samples from medical clinics (Dearwater et al., 1998), indicate that having children is a significant risk factor for partner violence, while others indicate that it is not (Lövestad & Krantz, 2012).

The role of children in the context of a divorce is also unclear in the research literature. Some studies have suggested that since women with children are more likely to stay in contact with their estranged husbands, they may therefore remain more at risk for partner violence and its threat after divorce (Brownridge, 2006). However, it cannot be completely ruled out that some aggressive men who face divorce refrain from partner violence out of fear that it would limit their access to their children. Furthermore, in a previous study based on a nationally representative life history survey conducted among ever‐divorced people in the Netherlands, Fischer et al. (2005) showed that former couples with joint children have more contact—both antagonistic and friendly—than couples without children. It has also been suggested that parenting may increase vulnerability to the negative effects of divorce (Amato, 2000; Williams & Dunne‐Bryant, 2006) and that specific conflicts about the children may lead to more general conflicts, especially when the children are young and less independent (Fischer et al., 2005). These conflicts could escalate into partner violence even after divorce.

The present study aims to assess whether the greatest risk of partner violence occurs before, during, or after divorce. Our hypothesis states that the highest risk occurs shortly before divorce when relationship conflicts have the highest potential to escalate. We also look at the risks according to whether divorcing women have children with their husbands. Our hypothesis for the mediating role of children postulates that women with children—especially younger children—face more partner aggression and its threats following divorce, given that they have more contact with their former spouses than women without children.

## DATA AND METHODS

### Data

We carry out a record‐linkage study originally based on the total population data of women aged 55 and under who ever resided in Finland in 2000–2015. We restrict the dataset to women born in 1960 or later and link them with their husbands and biological children using the unique pseudonymized identifiers, the household‐linkage tables, and the reproductive‐linkage tables provided by Statistics Finland. The pseudonymized identifiers—based on the country's 11‐digit personal identification codes—uniquely identify all citizens and individuals who live in Finland. In addition to information on marriages, divorces, and deaths, Statistics Finland also provided information on individuals' socio‐demographic characteristics. The National Institute for Health and Welfare provided information on hospital‐treated injuries (first outcome) and Statistics Finland provided information on police‐reported crimes (other outcomes). The register‐based study profiles describing how we constructed the population datasets for the analyses of hospitalizations and crimes are shown in Figure S1.

### Hospitalization for assault injuries: Study population and hospital diagnoses

We first focus on 41,766 married women who divorced between 2011 and 2014 and follow their outpatient and inpatient hospitalizations for assault injuries for 4 years before and 3 years after their divorce (2007–2017). If a study person experiences several divorces between January 1, 2011 and December 31, 2014, we use information on the most recent divorce. We complement these data for divorcing women with a comparison group of women who stay continuously married for the observation period. The comparison group includes 304,026 continuously married women who were married to and lived with their husbands for at least 2 years between December 31, 2012 and December 31, 2014, and who did not experience any divorce, separation, or widowhood between January 1, 2005 and December 31, 2017.

We use the National Hospital Discharge Records from 2006 to 2017 to form the outcome of outpatient or inpatient hospitalizations for assault injuries, using the Tenth Revision of the International Statistical Classification of Diseases and Related Health Problems (ICD‐10). The study comprises the external causes of the injuries, including both physical and sexual causes (ICD‐10 codes: X85–Y09). A few women who were killed without being hospitalized are included as cases in our outcome measure on the day of their death, based on their death records. The outcome is coded as one if a woman is hospitalized at least once during a 6‐month observation period and zero otherwise.

### Crime victimization: Study population and crime types

We then focus on 22,468 married women who divorced in 2014–2015 and follow their crime victimization for and perpetration of partner violence for 3 years before and 2 years after their year of divorce (2011–2017). We complement the data for divorcing women with a comparison group of women who stay continuously married for the observation period. The reason for limiting the timelines differently for crimes and hospitalizations is that medical records have been available for a long period of time but police records for petty assaults improved in 2011 following a legal reform. Since 2011, petty assaults could be prosecuted and investigated by the police without the victim's active request, resulting in the better coverage of partner assaults (Aaltonen et al., 2014). The dataset of the divorced for the analyses of crimes (*N* = 22,468) is smaller than that for hospitalizations (*N* = 41,766) because of the different timelines to collect divorces (2 vs. 4 years).

The comparison group includes 333,542 continuously married women who were married to and lived with their husbands for at least 2 years between December 31, 2013 and December 31, 2015, and who did not experience any divorce, separation, or widowhood between January 1, 2011 and December 31, 2017. If a divorcing woman experienced several divorces between 2014 and 2015, we use information on the most recent divorce. If a divorcing woman's husband had also divorced his previous wife between 2014 and 2015, we use information for the wife who divorced him last. Our analytical dataset for crimes uniquely identifies both members of the couple.

Statistics Finland provided annual information on police‐reported crimes, categorized as domestic violence in 2009–2017. To create the outcome for victimization for partner violence, we use police records from 2011 to 2017. The data are based on crimes reported to the police, but they did not necessarily lead to a criminal charges or convictions, which is why the offenders are referred to as suspects. The outcome data for victimization are linked to the couple data by using the pseudonymized identifiers of both the female victim and the suspected perpetrator. The perpetrator needs to be the same person with whom the woman's marriage ended—or continued—to qualify for the victim–perpetrator linkage.

In our study, we differentiate between crime victimization for partner assault and menace. These two outcomes are studied separately. Victimization for assault mainly includes the following crimes: petty assault, assault or its attempt, aggravated assault or its attempt, and attempted homicide causing bodily injuries but not leading to the partner's death. With few exceptions, the entire chapter 21 of the Finnish Criminal Code (Homicide and Bodily Injury) is included in our analyses (Ministry of Justice, 2015). Few homicides and bodily injuries that resulted in the partner's death are excluded. The reason for this is that information on completed homicides was not available to us due to national data limitations. This is unlikely to bias our results, given that completed homicides are rare in relation to assaults. For example, between 2015 and 2019, only 66 women were killed by their current or former spouse in Finland, whereas the corresponding figure for assaults and attempted homicides was 3433 in 2015 only (Tanskanen, 2020).

In our study, victimization for menace includes illegal menace (25:7§) and stalking (25:7a§). Illegal menace is punishable when “A person who raises a weapon at another or otherwise threatens another with an offence under such circumstances that the person so threatened has justified reason to believe that his or her personal safety or property or that of someone else is in serious danger shall be sentenced for menace…(Ministry of Justice, 2015).” Stalking became punishable in 2014 and is small in magnitude compared with illegal menace.

### Crime perpetration and mutual violence

We use the same dataset of the divorced for perpetration as for crime victimization (*N* = 22,468). We study the same crimes for women as perpetrators as for women as victims: partner assaults and menace. To qualify for the perpetrator–victim linkage, the perpetrator needs to be the wife and the victim the husband. Given that we had constructed two crime datasets for partner assaults for women—the first dataset for women as victims of their husband's violence and the second for women as perpetrators of the violence—we merged these datasets for divorcing women to study the extent to which the occurrence of police‐reported partner assault in a given year was mutual. In these analyses, we excluded the comparison group of continuously married women because of their extremely low crime rates against their husbands.

### Control variables

We use control variables measured for the women or the couple. The women's order of marriage is categorized into three groups: 1st, 2nd, and 3rd or higher order marriage. A few women whose order of marriage was not known are categorized as being married for the first time. The duration of marriage is categorized into six groups: 0–4 years, 5–9 years, 10–14 years, 15–19 years, 20–24 years, and 25 years or more. The number of biological children under the age of 18 is categorized into two groups: no children and one child or more. Education is categorized into three groups according to the highest educational qualification or degree completed by the women: basic education or less (typically 9 years of education), intermediate education (typically secondary‐level vocational school or academic upper secondary school degrees), and tertiary education (typically vocationally oriented polytechnic school degrees and bachelor's or master's degrees). Age is a variable with 1‐year increments, based on the year and month of birth. It is a time‐varying variable. Calendar year is used as a time‐varying variable in the analysis of hospitalization, but it is time‐invariant in the analysis of crimes due to its multicollinearity with years surrounding divorce year.

### Statistical analyses

**FIGURE 1:**
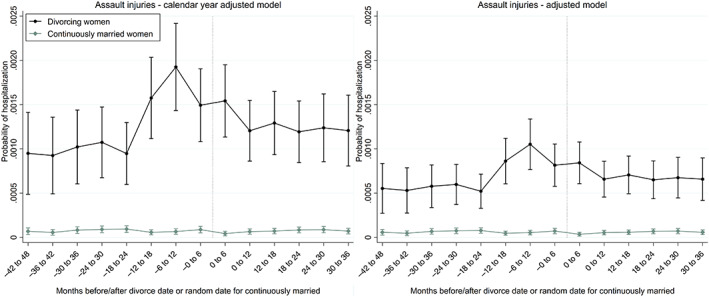
Trajectories of hospitalization for assault injuries (6‐month probability) before and after the date of divorce for divorcing women, and before and after a random date for continuously married women. The predicted probabilities shown in the left panel are adjusted for calendar year only and those in the right panel are adjusted for calendar year, age, education, the duration and order of marriage, and the number of children. Error bars are 95% confidence intervals. [Color figure can be viewed at wileyonlinelibrary.com]

We assess the trajectories of hospitalizations for assault injuries for 4 years before and 3 years after the date of divorce. We divide the time to and since divorce into 14 six‐month (183‐day) observation periods surrounding the date of divorce for divorcing women and surrounding a random date—randomized between January 1, 2013 and December 31, 2014—for the continuously married. We use repeated‐measures logistic regression analysis with an outcome of one if a woman received inpatient or outpatient hospital care for assault injuries at least once during a 6‐month observation period and zero otherwise. The analysis controls for correlations within individuals by using generalized estimation equations with an unstructured correlation matrix (Lipsitz et al., 1994; Twisk, 2003). The results are shown in Figure 1 as calendar year‐adjusted predictions (left panel), and as adjusted predictions estimated at the mean value of other observed covariates (right panel). The adjusted predictions are controlled for time‐varying covariates of calendar year and age, and for time‐invariant covariates of education, the duration and order of marriage, and having children. The adjusted predictions can be interpreted as the predicted probabilities of hospitalization for divorcing and continuously married women if they all were average in all other respects.

The follow‐up time is left‐censored preceding the period in which the union started by using information on wedding dates and cohabitation starting dates, whichever came first. The reason for this is that the couple needed to be together in order to contribute to the pre‐divorce estimates. The follow‐up time is right‐censored in the next period following death or December 31, 2017 at the latest. The average follow‐up time is 6.91 years for divorcing women and 6.97 years for continuously married women. Table 2 displays information on the number of persons included and hospitalized at each observation period.

We assess the trajectories of crime victimization for partner assault and menace, using the same approach—repeated‐measures logistic regression analysis. We divide the time to and since divorce into 6 annual observation windows surrounding the year of divorce for divorcing women and surrounding the year 2015 for continuously married women (−3, −2, −1, 0, +1, +2). The outcomes are defined as one if women are victimized at least once during the observation window and zero otherwise. The results are presented as unadjusted predictions (left panel) and as adjusted predictions, estimated at the mean value of other covariates (right panel). The adjusted predictions are controlled for time‐varying covariates of age, and for time‐invariant covariates of year, education, the duration and order of marriage, and having children. Table 1 contains the descriptive statistics of the covariates. Table 3 displays information on the number of persons included and victimized at each observation period.

**TABLE 1:** Characteristics of women who were continuously married or who experienced divorce between 2011 and 2014 for the analysis of hospitalization for assault injuries (hospital datasets), and characteristics of women who were continuously married or who experienced divorce between 2014 and 2015 for the analyses of crime victimization and perpetration, Finland

Characteristic	Hospital datasets		Crime datasets	
	Divorcing women (*N* = 41,766) %	Continuously married women (*N* = 304,026) %	Divorcing women (*N* = 22,468) %	Continuously married women (*N* = 333,542) %
Age				
Mean	38.5	41.6	39.6	43.1
Standard deviation	7.9	8.1	8.2	8.3
Order of marriage				
First	83.6	92.3	83.3	91.0
Second	14.2	7.1	14.4	8.1
Third or more	2.2	0.6	2.3	0.8
Duration of marriage (years)				
0–4	23.2	12.9	21.6	11.2
5–9	30.3	20.6	31.3	21.1
10–14	18.4	17.8	19.1	17.8
15–19	12.7	15.3	12.1	14.7
20–24	9.2	15.0	9.0	14.1
25 or more	6.1	18.4	6.9	21.0
Having children				
No children	39.6	43.6	40.4	45.7
One child or more	60.4	56.4	59.6	54.3
Education level				
Basic or less	12.8	6.7	12.5	7.1
Intermediate	43.6	36.1	43.0	35.8
Tertiary	43.6	57.2	44.5	57.1
Total	100.0	100.0	100.0	100.0

We assess the victimization trajectories of partner assault (left panel) and menace (right panel), according to whether women have children. These results are presented as the unadjusted trajectories for divorcing and continuously married women (Figure 4). We then exclude the comparison group of the married and assess the adjusted trajectories for divorcing women by the age of their youngest child or childlessness (Figure 5). We use Wald tests to examine whether at a particular observation period there is a difference in the adjusted probability of victimization between divorcing women with no children and those with younger or older children (Table S1). *P*‐values for these differences are based on the standard errors calculated using the delta method. Table S1 also shows whether the change in the probability, relative to the first observation period of 3 years before divorce, is different between these groups of divorced women.

To study the trajectories of crime perpetration by women against their husbands, we use the same longitudinal approach. These findings are presented as the unadjusted trajectories for divorcing women alongside the corresponding figures for victimization, to illustrate the level differences observed for women surrounding divorce. We also describe the number of women who are only victims, both victims and perpetrators, and only perpetrators in a given year surrounding divorce.

### Children

To study whether having children modified the trajectories of crime victimization, we first categorize having biological children into two groups: no children and one child or more. Having children aged under 18 years is defined by using the pseudonymized identifiers of both the mother and the father and using information on their children's vital statuses and ages. Children who died or reached the age of 18 before the month of divorce are excluded from the total sum of children, as well as those who were not fathered by the husband their mother divorced. Secondly, we further categorize women according to the age of their youngest child as follows: no children, youngest child aged under 12 years, and youngest child aged 12 years and over.

## RESULTS

### Assault injuries

Table 1 displays the characteristics of the study populations for the analyses. Divorcing women were younger, had fewer children and marriages of higher order and shorter duration than those who were continuously married. Table 2 shows the total number of persons observed and hospitalized in each 6‐month observation period surrounding a divorce date for divorcing women and a random date for continuously married women. Of the divorcing women, 1.27% had at least one hospital admission for assault injuries during the 7‐year follow‐up time. The corresponding figure for continuously married women was only 0.09% (Table 2). However, it is noteworthy that these figures include women who could have been victimized multiple times during the follow‐up time.

**TABLE 2:** The number of persons at each 6‐month observation period surrounding the date of divorce for divorcing women and a random date for continuously married women, and the number and percentage of persons who experience hospitalization for assault injuries, Finland

Hospital dataset	Persons (*N*)	Hospitalization for assault injuries (*N*)	(%)
*Divorcing women*			
Time before/after the date of divorce (months)			
−40 to −46	38,738	22	0.06
−36 to −40	39,624	24	0.06
−30 to −36	40,388	30	0.07
−24 to −30	41,063	35	0.09
−18 to −24	41,503	34	0.08
−12 to −18	41,724	58	0.14
−6 to −12	41,766	72	0.17
−0 to −6	41,766	57	0.14
0 to 6	41,766	60	0.14
6 to 12	41,737	50	0.12
12 to 18	41,723	57	0.14
18 to 24	41,693	56	0.13
24 to 30	41,681	62	0.15
30 to 36	41,660	63	0.15
Ever hospitalized during the 7‐year follow‐up time		529	1.27
*Continuously married women*			
Time before/after a random date (months)			
−40 to −46	297,558	16	0.01
−36 to −40	299,443	14	0.00
−30 to −36	301,027	22	0.01
−24 to −30	302,280	25	0.01
−18 to −24	303,195	27	0.01
−12 to −18	303,707	16	0.01
−6 to −12	303,978	18	0.01
−0 to −6	304,026	24	0.01
0 to 6	304,026	12	0.00
6 to 12	304,012	21	0.01
12 to 18	303,963	26	0.01
18 to 24	303,911	34	0.01
24 to 30	303,820	39	0.01
30 to 36	303,704	31	0.01
Ever hospitalized during the 7‐year follow‐up time		268	0.09

For divorcing women, the risk of assault injuries was already elevated at baseline 4 years before divorce, as compared with continuously married women (Figure 1, left panel). The risk depended on the time to divorce and increased more around 18 months before divorce. The risk, adjusted for calendar year only, peaked from 6 to 12 months before divorce when approximately 0.2% of divorcing women were medically treated for their assault injuries in a 6‐month observation period. The risk—both calendar year‐adjusted and fully adjusted—decreased following divorce and appeared to return to levels observed 3–4 years before divorce. However, their risk stayed at a heightened level following divorce, as compared with those who are continuously married.

Given that the risk of assault injuries for the divorced did not reach the low levels of the continuously married after divorce, we provided a sensitivity analysis (Figure S2), in which the hospital outcome was recorded from one to zero throughout the follow‐up time for 2 years before and 3 years after divorce if the husband was never reported to the police for assaulting this wife between 2009 and 2017. The idea is to provide a conservative trajectory, in which the husband could be the perpetrator only if the police had ever registered him as such against this wife. Of the divorcing women who were medically treated for assault injuries during the follow‐up time of 2 years before and 3 years after divorce, 41% were registered victims of their husbands in the police‐reported crime files of domestic violence. It is possible that the rest of the women who received treatment for their injuries were injured by persons other than the husband or did not report partner assault to the police. The corresponding coverage for continuously married injured women was 26%, providing indirect support for the idea that married women might have stronger intentions to protect the perpetrator. Figure S2 indicates that the risk of assault injuries rapidly leveled off following divorce if only the wives of the police‐registered perpetrators contributed to the outcome. In any case, our sensitivity analysis confirms that the peak in assault injuries is from 6 to 12 months prior to divorce.

Had we used the date of separation, based on living togetherness, as our marital status transition point for women who divorced*—*instead of the official divorce date—the findings would not have substantially changed. Our sensitivity analysis confirms that the highest risk for divorcing women is before the breakup, regardless of whether assault injuries are analyzed around the separation date or divorce date (Figure S3). Given the lag between separation and divorce (mean ~ 170 days), the highest peak in assault injuries occurred from 1 to 183 days (0–6 months) before separation, whereas the highest peak surrounding divorce occurred from 184 to 366 days (6–12 months) before divorce (Figure 1).

### Crime victimization

Table 1 displays the characteristics of the study population for the analysis of crime. Table 3 displays the total number of persons observed and crime victimized in each annual observation period surrounding the year of divorce. Of the 22,468 divorcing women, 4.5% were ever crime victimized for partner assault by their husbands during the 6‐year follow‐up time. The corresponding figure for continuously married women was only 0.3% (Table 3).

**TABLE 3:** The number of persons at each annual observation period surrounding the year of divorce for divorcing women and 2015 for continuously married women, and the number and percentage of persons who experience crime victimization for partner assault or menace, Finland

Crime datasets	Persons (*N*)	Crime victimization for partner assault (*N*)	(%)	Crime victimization for menace (*N*)	(%)
*Divorcing women*					
Time before/after the year of divorce (years)					
−3	21,802	221	1.01	48	0.22
−2	22,355	247	1.10	60	0.27
−1	22,467	470	2.09	174	0.77
0	22,468	223	0.99	148	0.66
+1	22,453	64	0.29	49	0.22
+2	22,453	48	0.21	31	0.14
Ever victimized during the 6‐year follow‐up time		1013	4.51	461	2.05
*Continuously married women*					
Time before/after 2015 (years)					
−3	331,872	241	0.07	35	0.01
−2	333,542	195	0.06	19	0.01
−1	333,542	186	0.06	20	0.01
0	333,542	183	0.05	20	0.01
+1	333,542	171	0.05	29	0.01
+2	333,291	183	0.05	41	0.01
Ever victimized during the 6‐year follow‐up time		1024	0.31	162	0.05

**FIGURE 2:**
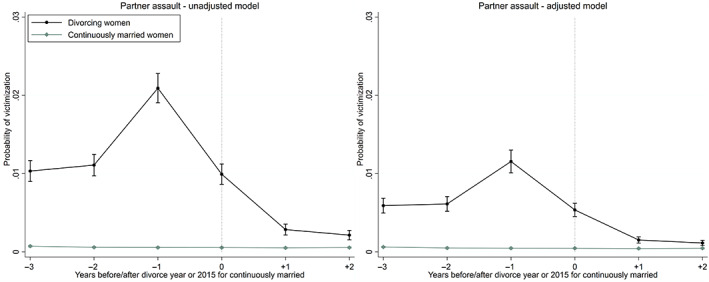
Trajectories of crime victimization for partner assault (annual probability) before and after the year of divorce for divorcing women, and before and after the year 2015 for continuously married women. The predicted probabilities shown in the left panel are unadjusted and those in the right panel are adjusted for year, age, education, the duration and order of marriage, and the number of children. Error bars are 95% confidence intervals. [Color figure can be viewed at wileyonlinelibrary.com]

Figure 2 shows that the risk of victimization for partner assault was already higher for divorcing than continuously married women from 2 to 3 years before divorce. The risk further increases in the year prior to the year of divorce. Two out of one hundred divorcing women were physically assaulted by their husbands in their last full marital year, as measured by violence reported to the police (Figure 2, left panel). However, the risk decreased with time since divorce and reached the low levels of the continuously married from 1 to 2 years following divorce—especially when sociodemographic differences between the two groups were controlled for (Figure 2, right panel).

**FIGURE 3:**
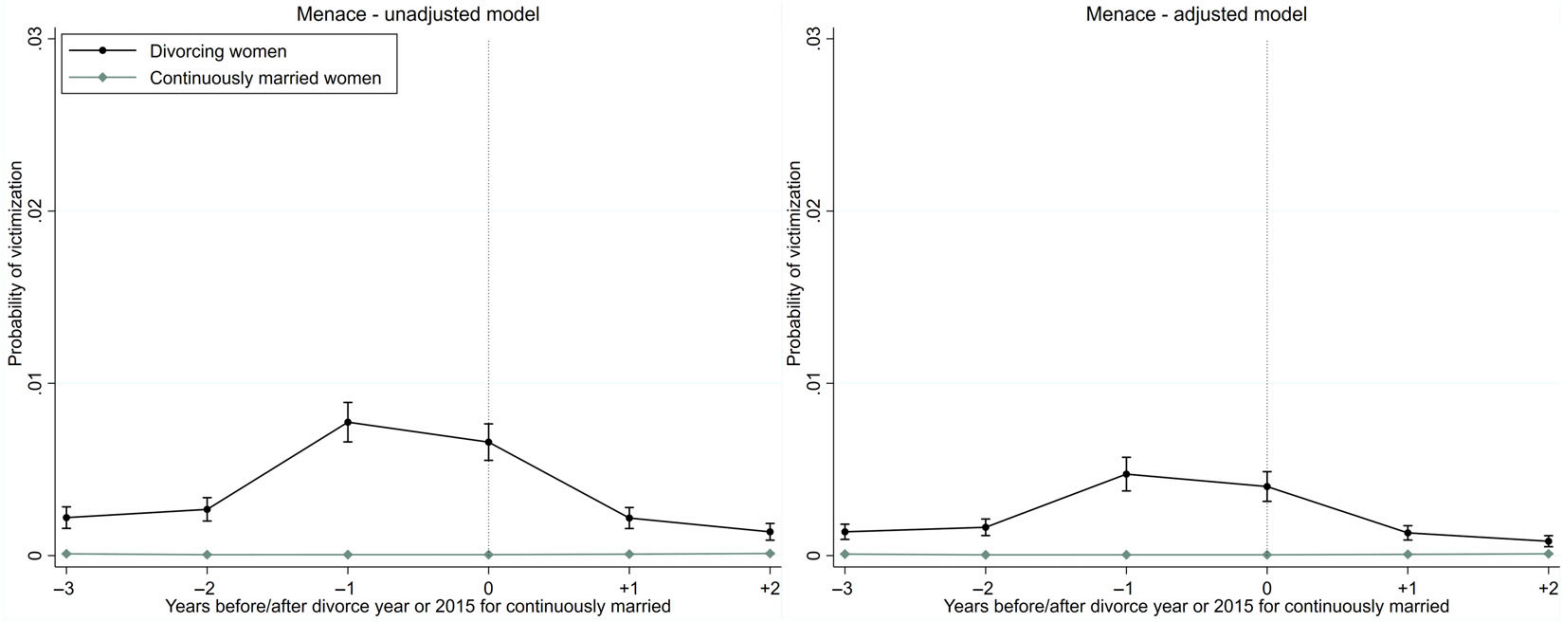
Trajectories of crime victimization for menace (annual probability) before and after the year of divorce for divorcing women, and before and after the year 2015 for continuously married women. The predicted probabilities shown in the left panel are unadjusted and those in the right panel are adjusted for year, age, education, the duration and order of marriage, and the number of children. Error bars are 95% confidence intervals. [Color figure can be viewed at wileyonlinelibrary.com]

Figure 3 shows that the risk of crime victimization for menace was already higher for divorcing than continuously married women 2–3 years prior to divorce. The risk further increased in the year prior to and in the year of divorce. Approximately 0.8% of divorcing women were victimized for menace by their husbands in the year prior to the year of divorce, as measured by the women reporting the threat to the police (left panel). The risk decreased rapidly with time since divorce and reached the low levels of the continuously married after 2 years following divorce—particularly according to the model that controlled for sociodemographic differences between divorcing and continuously married women (right panel).

### Crime victimization for women with and without children

**FIGURE 4:**
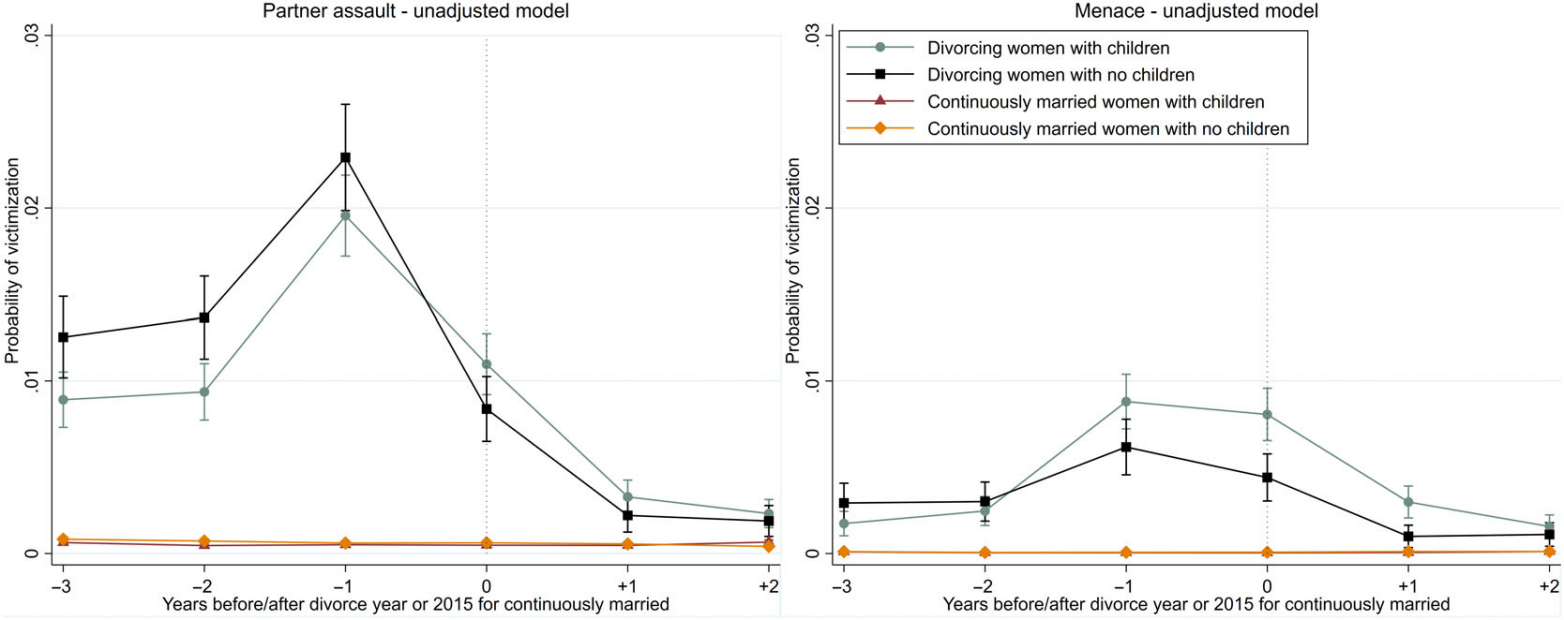
Unadjusted trajectories of crime victimization for partner assault (left panel) and menace (right panel) before and after the year of divorce for divorcing women, and before and after the year 2015 for continuously married women, according to whether women have children with their husbands. Error bars are 95% confidence intervals. [Color figure can be viewed at wileyonlinelibrary.com]

Figure 4 indicates that the unadjusted trajectories of crime victimization for physical partner assault are relatively similar, regardless of whether the divorcing couple has children. Women with children appear to have a somewhat lower risk of victimization for partner assault from 1 to 2 years prior to the year of divorce, compared with divorcing women without children (left panel). In the year of divorce, women with children had the same unadjusted risk as women without children.

**FIGURE 5:**
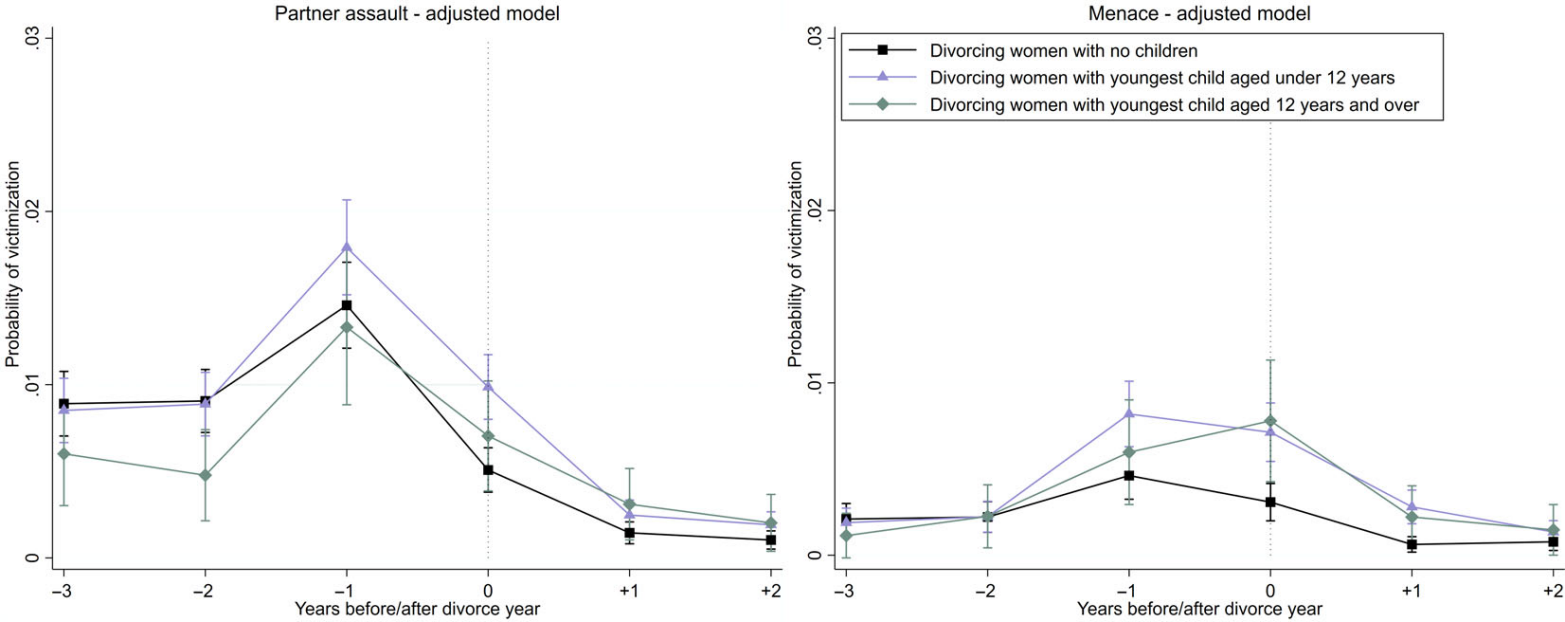
Trajectories of crime victimization for partner assault (left panel) and menace (right panel) before and after the year of divorce for divorcing women, according to the age of the youngest child. The predicted probabilities are adjusted for year, age, education, and the duration and order of marriage. Error bars are 95% confidence intervals. [Color figure can be viewed at wileyonlinelibrary.com]

Figure 5 reveals that the risk of assault depends on the age of the youngest child (left panel). Women with younger children aged under 12 years had a significantly higher risk of assault in the year before and in the year of divorce, compared with women with no children (Table S3). On the contrary, women with older children aged 12 years and over had the same risk than women with no children in the year before and in the year of divorce. In addition, women with older children had a lower risk than childless women from 2 to 3 years prior to divorce.

Figure 4 shows that the trajectories of menace depend on whether divorcing women have children (right panel). First, the risk of menace was the same from 2 to 3 years prior to divorce, then it increased more for women with children, and was significantly higher for women with children in the year of divorce. The excess risk for women with children persisted for 1 year following the year of divorce, and then leveled off after 2 years since divorce.

Figure 5 shows that women with younger children aged under 12 years as well as women with older children aged 12 years and over had elevated risks of menace in the year of and in the year after divorce, compared with women without children (right panel). For women with younger children, the risk was significantly elevated already in the year before divorce (Table S3). The excess risks leveled off after 2 years since divorce.

### Crime perpetration and mutual partner assaults among divorcing women

**FIGURE 6:**
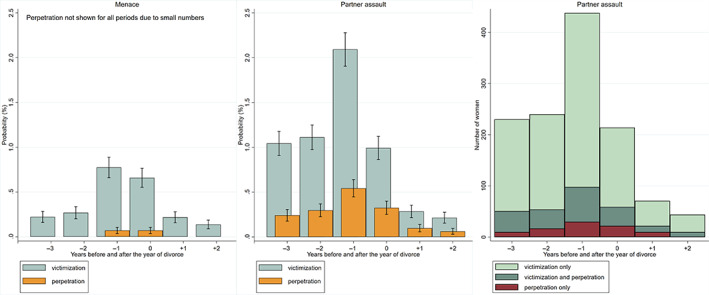
Unadjusted trajectories of crime victimization for and perpetration of menace (left panel) and of partner assault (middle panel) for divorcing women, and the number of divorcing women who experienced partner assault as a victim only, as a victim and perpetrator, and as a perpetrator only before and after the year of divorce (right panel). Error bars are 95% confidence intervals based on the predicted probabilities from repeated‐measures logistic models. [Color figure can be viewed at wileyonlinelibrary.com]

To shed light on mutual partner violence, we studied the unadjusted trajectories of perpetration of partner violence alongside the corresponding victimization trajectories for divorcing women. Figure 6 shows that the risk of perpetration of partner assault (middle panel) and menace (left panel) was substantially lower in magnitude than that of victimization. There was, however, an increasing risk of perpetration in the year prior to the year of divorce. As shown in Figure 6 (right panel), the majority of partner assault cases in which women are perpetrators are mutual, in the sense that the husband has also physically assaulted the wife. Of the 122 women perpetrators of partner assault who were reported to the police in the year prior to the year of divorce, 73% were also physically assaulted by their husbands in the same year. Had we taken into account 2‐year lags, the proportion would have risen to 79%. The rest of women perpetrators were not victimized for assault by their husbands, as measured by incidences reported to the police.

## DISCUSSION

### Divorcing women as victims

Our findings for divorcing women showed that the risk of hospitalization for assault injuries was already elevated from 3 to 4 years before divorce, compared with women who were continuously married. The risk further increased with an approaching divorce and was particularly elevated from 6 to 12 months before divorce. There are several complementary explanations for this observation. First, women who are physically assaulted to the extent that they need medical treatment for their assault injuries might decide to file for divorce after the incident. This argument is in line with previous studies indicating that partner violence and its severity are related to an increased risk of marital dissolution (Kingston‐Riechers, 2001). Injured women may receive support from their relatives or hospital staff to make the decision to divorce. In Finland, a divorce can be granted after a reconsideration period of 6 months (Ministry of Justice, 2002), and the timeline of the greatest risk coincides with the idea that physically injured women file for divorce.

Secondly, the time of the greatest risk for assault injuries also coincides with the idea that quarrels escalate into violence once one member of the couple discloses his or her divorce intentions. In Finland as in the United States, women are more likely than men to initiate divorce (Rosenfeld, 2018). This argument of the dangers of the start of a divorce process is partly in line with prior data on homicides, indicating that the period shortly after separation, but before the official divorce, is the time of the greatest risk for post‐separation violence in certain countries (Brownridge, 2006). In his literature review of post‐separation violence, Brownridge (2006) summarized partly based on the study of Hotton (2001) that “Data from Canada, the United States, New South Wales, and Australia indicate that about half of intimate femicides by ex‐partners occur within 2 months following separation (Hotton, 2001).” It is thus possible that the highest peak observed in our data, from 6 to 12 months prior to divorce, is not only caused by situational couple violence, escalating from relationship quarrels, but also by coercive control violence, by which the husband tries to regain control over his wife in order to prevent the divorce. It has been suggested that the subjective benefits of using partner violence (e.g., getting the wife to change her mind) are larger when the divorce is still an open question (Ekbrand, 2006). Our study indicated that the risk of assault injuries peaked from 6 to 12 months before divorce when most women were still living with their husbands (76%), and therefore vulnerable for abuse.

Our findings for crime victimization for partner assault confirmed the results observed for hospital‐treated assault injuries. The risk of partner assault increased with an approaching divorce and peaked in the year prior to the year of divorce, and then decreased with time since divorce. However, it cannot be completely ruled out that police‐reported crimes could be subject to self‐reporting bias, particularly in the context of an approaching divorce. Women who have experienced violence during their marriage and have finally decided to end their marriage might no longer avoid contact with the police in order to protect the perpetrator. Qualitative evidence suggests that partner violence is often disclosed to other people out of the necessity to end the violent situation. Partner violence can be hidden for a long time because of its stigma and related feelings of guilt, fear, and desires to protect the perpetrator (Boethius & Åkerström, 2020). Its disclosure to the police in the year prior to divorce could be motivated by an increased need for help that exceeds the need to protect the perpetrator. It is well‐known that not all victims report partner violence to the police, but reporting becomes more likely when physical violence is more severe (Heiskanen & Ruuskanen, 2010). It is clear, however, that police‐reported violence underestimates the true incidence of partner violence. For example, Finnish cross‐sectional surveys on victimization from 2013 to 2019 indicate that approximately 6% of all women have experienced partner violence or its threat by their current or former spouses within the past year (National Institute for Health and Welfare, 2021). Our study indicates that only 2% of divorcing women experienced police‐reported partner assault in the year prior to the year of divorce when the annual risk was especially elevated.

It is noteworthy that a register‐based observational study, such as ours, that measures the violence before and after divorce does not differentiate between the extent to which the violence causes divorce and the extent to which the announcement to divorce causes violence. Survey‐based approaches could better capture when the decision to divorce was announced to the spouse and its causal effects on the violence.

We also studied crime victimization for partner assault and menace according to whether the couple had children together. Divorcing women with children were more likely to experience menace in the year of and in the year after the year of divorce. The result is in line with the idea that women with children remain more at risk for partner violence (Brownridge, 2006)—particularly menace—when the couple stays in contact with each other because of their children. This finding was observed for both women with younger and older children. This is an alarming finding given that abused women who experience psychological partner violence are at a heightened risk of developing posttraumatic stress disorders (Pico‐Alfonso, 2005), and that these could be more prevalent in women who need to take care of children.

The analysis of physical violence suggested that women with younger children aged under 12 years experienced an elevated risk of assault in the year before and in the year of divorce, compared with women with no children. A similar pattern of findings was not observed for women with older children. These results are in part consistent with the idea that specific conflicts about the children may lead to more general conflicts, especially when the children are young (Fischer et al., 2005).

### Divorcing women as perpetrators

One of the unique features of our study is that the crime data enabled us to study whether and to what extent women physically assaulted their husbands in the context of a divorce. Our study indicated that women's risk of crime perpetration is substantially lower than their risk of victimization. There is, however, an increasing risk of perpetration during the year prior to the year of divorce for women as well. The majority of these cases are mutual, in the sense that the husband has also physically assaulted the wife. Based on the theory of control violence (Johnson, 2008), it is possible that mutual violence is resistant violence that arises in reaction to the husband's use of violence. Prior qualitative evidence suggests that women as perpetrators of violence often link the beginning of their own use of violence to long‐term victimhood in intimate relations (Venalainen, 2017). Of course, it is also possible that mutual violence observed in our study is a physical fight between the two equal members of the couple or that the resister is the husband. Few prior population‐based studies have looked at victimization and perpetration combined. In a population‐based cross‐sectional study of 173 Swedish men and 251 women, Lövestad and Krantz (2012) found that a considerable proportion of the men victimized for partner violence also used such violence, while this was less common in victimized women. The result is in line with our finding, although it is representative of Swedish population of age 18–65, and not particularly those who divorce.

In our study, the risk of perpetration of menace is especially low among women, although its peak is visible in the year prior to the year of divorce. It is possible that threatening to seriously injure or kill one's partner is considered societally particularly unsuitable for women to do, even in the context of an escalating situation. However, it is also possible that men do not feel that threatened by their wives' threats, in the sense that they would feel the need to report them to the police. There is prior evidence to suggest that since women mostly are physically disadvantaged, compared with men, they are more likely to recall and report past violent incidents and to feel more threatened (Lövestad & Krantz, 2012). Of course, it is also possible that men feel that the police would not take them seriously.

### Strengths and limitations

The main strength of our study is that Statistics Finland pseudonymized multiple datasets for our research purposes by replacing the 11‐digit personal identification codes of the members of the couple and their children by artificial identifiers. These artificial identifiers allowed us to build record‐linkage trajectories of hospital‐treated assault injuries and police‐reported partner violence surrounding divorce. We could also assess whether the victimization trajectories were similar for divorcing women with and without children. The record‐linkage data are well‐suited for longitudinal study designs and have minimal loss to follow‐up. The datasets are representative of the divorcing population across all social strata. We also studied both crime victimization and perpetration allowing to shed light on mutual partner violence in the context of divorce.

One of the strengths but also a limitation of the study is that we used different outcome indicators for partner violence, including police‐reported partner assaults (suspected crimes) and hospital‐treated assault injuries (medical consequences). These outcome indicators provided partly similar and partly different results. Both indicators suggested that there is an increasing pattern of partner assault for women prior to divorce. However, the results for police‐reported assaults indicated that partner violence decreased rapidly from 1 to 2 years after divorce, indicating that most aggressive ex‐husbands adjusted to life post‐divorce (Figure 2). On the contrary, the risk of being medically treated for assault injuries did not reach the low levels of the continuously married after 2 years following divorce (Figure 1). There are several possible explanations for this partial inconsistency observed for the two different types of outcomes. First, it is possible that women who never report partner violence to the police are assaulted to a higher extent following divorce than their counterparts who report violence to the police. Second, medical care can be caused by past injuries that are repeatedly cared for, whereas a crime is registered only once. Thirdly, the crime data assure that the perpetrator is always the same husband, while different perpetrators can cause hospital‐treated injuries. There is, however, prior evidence from England to suggest that the most likely perpetrator of adult women is an intimate partner, rather than an acquaintance or a stranger (Office for National Statistics, 2019). In victimization surveys from Finland, however, women report relatively often that they have also been assaulted or threatened by a stranger or an acquaintance that they barely knew (National Institute for Health and Welfare, 2021). On the other hand, homicide data for Finnish women show that the perpetrator is often a current or former husband in crimes leading to the victim's death. Between 2003 and 2013, over 65% of women who were killed in Finland were killed by a current or former husband, a common‐law husband, or a dating partner (Lehti, 2015).

In our hospitalization analyses, we used the 3‐digit ICD‐10 codes for assaults (ICD‐10: X85–Y09), given that the perpetrator identifiers were not widely used for medical data at the time of our study (Kivelä et al., 2019). To correct for possible bias arising from wrongly labeling assaults as partner assaults, Figure S2 showed a sensitivity analysis that excluded all events for those women whose husbands had not been reported to the police for partner violence against this wife. It is, however, noteworthy that our sensitivity analysis might have excluded too many cases because not all seriously injured women report partner violence to the police. It is also possible that several cases of partner violence are misclassified as accidents. There is prior evidence to suggest that women's facial bone fractures—a rather typical consequence of serious partner violence (Le et al., 2001)—are disproportionally large in fall‐related injuries treated in hospitals, compared with men (Kontio, 2002). There is no reason to believe that women would experience more facial bone fractures than men from fall‐related injuries, unless part of them were assault injuries, possibly caused by their partners.

One of the limitations of our study is that we were unable to study crime victimization for partner assaults surrounding separation from nonmarital cohabitation. The reason for this is that the crime data of domestic violence are not longitudinally available for nonmarried women after cohabitation with their common‐law husband ends. This is unfortunate because cohabitations are increasingly common and less stable than marriages (Jalovaara, 2013; Jalovaara & Kulu, 2018). In 2013, for example, women's cohabitations outnumbered marriages among 20‐ to 24‐year‐olds (84%) and 25‐ to 29‐year‐olds (58%), but not among 30‐ to 34‐year‐olds (36%) or 35‐ to 39‐year‐olds (26%) (Mäenpää, 2015, p. 23). Furthermore, our data did not include information on the member of the couple who filed for divorce. It has been suggested that a breakup initiated by a wife could be more dangerous for women, given that some men may use partner violence in an attempt to prevent divorce (García‐Ramos, 2021). Another limitation is that dating violence cannot be captured with register‐linkage studies, such as ours. Therefore, large‐scale surveys with meticulous follow‐up designs on dating violence observed before and after breakup are needed.

To summarize, using record‐linkage data on police‐reported crimes and hospital‐treated assault injuries to construct the trajectories of victimization for intimate partner violence before and after divorce, we showed that the risk of victimization is highest in the last marital year prior to divorce. The findings suggest that the time of the greatest risk is in the period in which the divorce petition is filed for. The timing coincides with the idea of violence aggravating alongside the decision to divorce but also with women's decision to divorce because of the violence. These findings are not only scientifically important but also practically relevant, as they could provide guidance in improving risk assessment tools and help to allocate spare resources of social workers, police officers, and multiagency teams—for example, to protect women with a history of partner violence who try to divorce. Victim questionnaires on intentions and attempts to divorce could be more widely used in developing risk assessment tools to protect the victims. Our findings indicate that divorcing women also perpetrate violence against their husbands during the transition to divorce, but its occurrence is smaller in magnitude and the violence is often accompanied by men's violence.

Our results indicate that divorcing women with younger children aged under 12 years have an elevated risk of victimization for physical assault in the year before and in the year of divorce. Furthermore, women with both younger and older children remain more at risk for menace for a year after the year of divorce, indicating that psychological violence is more likely to continue when the members of the couple stay in contact with each other because of their children. Psychological violence, which causes no bodily injuries but leads to enormous stress, could serve the purpose of retaliation and revenge after divorce. This phenomenon of psychological violence needs to be investigated further in future studies, given that it could affect the victim's quality of life following divorce.

## Supporting information


**Table S1** Trajectories of crime victimization for partner assault and menace before and after divorce for divorcing women without children, divorcing women with the youngest child aged under 12 years, and divorcing women with the youngest child aged 12 years and over, adjusted predicted probabilities, difference in probabilities, and difference in changes in probabilities, Finland.
**Figure S1**. Register‐based study profile describing the selection of women in the hospitalization datasets of divorced (*N* = 41,766) and continuously married women (*N* = 304,026) and in the crime datasets of divorced (*N* = 22,468) and continuously married women (*N* = 333,542)
**Figure S2**. Trajectories of hospitalization for assault injuries (6‐month probability) before and after the date of divorce for divorcing women, and before and after a random date for continuously married women. The outcome is “corrected” to zero for those whose husband is never registered as a perpetrator against this wife by the police from 2009 to 2017.
**Figure S3**. Trajectories of hospitalization for assault injuries (6‐month probability) before and after the date of separation (the end of cohabitation) for divorcing women, and before and after a random date for continuously married women.

## References

[jomf12881-bib-0001] Aaltonen, M. , Salmi, V. , & Kivivuori, J. (2014). Examining offender characteristics in police‐recorded domestic violence before and after a legal reform. International Criminal Justice Review, 24(3), 271–284. 10.1177/1057567714548454

[jomf12881-bib-0002] Afifi, T. O. , Cox, B. J. , & Enns, M. W. (2006). Mental health profiles among married, never‐married, and separated/divorced mothers in a nationally representative sample. Social Psychiatry and Psychiatric Epidemiology, 41(2), 122–129. 10.1007/s00127-005-0005-3 16467954

[jomf12881-bib-0003] Almond, L. , McManus, M. , Brian, D. J. , & Merrington, D. P. (2017). Exploration of the risk factors contained within the UK's existing domestic abuse risk assessment tool (DASH): Do these risk factors have individual predictive validity regarding recidivism? Journal of Aggression, Conflict and Peace Research, 9, 58–68.

[jomf12881-bib-0004] Amato, P. R. (2000). The consequences of divorce for adults and children. Journal of Marriage and Family, 62(4), 1269–1287. 10.1111/j.1741-3737.2000.01269.x

[jomf12881-bib-0005] Bernards, S. , & Graham, K. (2013). The cross‐cultural association between marital status and physical aggression between intimate partners. Journal of Family Violence, 28(4), 403–418. 10.1007/s10896-013-9505-1 24039342 PMC3769183

[jomf12881-bib-0006] Boethius, S. , & Åkerström, M. (2020). Revealing hidden realities: Disclosing domestic abuse to informal others. Nordic Journal of Criminology, 21, 186–202. 10.1080/2578983X.2020.1787725

[jomf12881-bib-0007] Bowlus, A. J. , & Seitz, S. (2006). Domestic violence, employment, and divorce. International Economic Review, 47(4), 1113–1149.

[jomf12881-bib-0008] Breslau, J. , Miller, E. , Jin, R. , Sampson, N. A. , Alonso, J. , Andrade, L. H. , Bromet, E. J. , de Girolamo, G. , Demyttenaere, K. , Fayyad, J. , Fukao, A. , Gălăon, M. , Gureje, O. , He, Y. , Hinkov, H. R. , Hu, C. , Kovess‐Masfety, V. , Matschinger, H. , Medina‐Mora, M. E. , … Kessler, R. C. (2011). A multinational study of mental disorders, marriage, and divorce. Acta Psychiatrica Scandinavica, 124(6), 474–486. 10.1111/j.1600-0447.2011.01712.x 21534936 PMC4011132

[jomf12881-bib-0009] Brownridge, D. A. (2006). Violence against women post‐separation. Aggression and Violent Behavior, 11(5), 514–530. 10.1016/j.avb.2006.01.009

[jomf12881-bib-0010] Brownridge, D. A. , Chan, K. L. , Hiebert‐Murphy, D. , Ristock, J. , Tiwari, A. , Leung, W.‐C. , & Santos, S. C. (2008). The elevated risk for non‐lethal post‐separation violence in Canada: A comparison of separated, divorced, and married women. Journal of Interpersonal Violence, 23(1), 117–135. 10.1177/0886260507307914 18087035

[jomf12881-bib-0011] Butterworth, P. , & Rodgers, B. (2008). Mental health problems and marital disruption: Is it the combination of husbands and wives' mental health problems that predicts later divorce? Social Psychiatry and Psychiatric Epidemiology, 43(9), 758–763. 10.1007/s00127-008-0366-5 18478168

[jomf12881-bib-0012] Catalano, S. , Smith, E. , Snyder, H. , & Rand, M. (2009). Female victims of violence. Bureau of Justice Statistics. Selected findings. U.S. Department of Justice, Office of Justice Programs, Bureau of Justice Statistics.

[jomf12881-bib-0013] Dearwater, S. R. , Coben, J. H. , Campbell, J. C. , Nah, G. , Glass, N. , McLoughlin, E. , & Bekemeier, B. (1998). Prevalence of intimate partner abuse in women treated at community hospital emergency departments. JAMA, 280(5), 433–438. 10.1001/jama.280.5.433 9701078

[jomf12881-bib-0014] Ekbrand, H. (2006). Separationer och mäns våld mot kvinnor (separations and men's violence against women). Göteborg University, Göteborg Studies in Sociology No 28.

[jomf12881-bib-0015] Fischer, T. F. C. , de Graaf, P. M. , & Kalmijn, M. (2005). Friendly and antagonistic contact between former spouses after divorce: Patterns and determinants. Journal of Family Issues, 26(8), 1131–1163. 10.1177/0192513X05275435

[jomf12881-bib-0016] García‐Ramos, A. (2021). Divorce laws and intimate partner violence: Evidence from Mexico. Journal of Development Economics, 150, 102623. 10.1016/j.jdeveco.2020.102623

[jomf12881-bib-0017] Garvin, V. , Kalter, N. , & Hansell, J. (1993). Divorced women: Individual differences in stressors, mediating factors, and adjustment outcome. The American Journal of Orthopsychiatry, 63(2), 232–240. 10.1037/h0079416 8484429

[jomf12881-bib-0018] Gerstel, N. , Riessman, C. K. , & Rosenfield, S. (1985). Explaining the symptomatology of separated and divorced women and men: The role of material conditions and social networks. Social Forces, 64(1), 84–101. 10.1093/sf/64.1.84

[jomf12881-bib-0019] Graham, L. M. , Sahay, K. M. , Rizo, C. F. , Messing, J. T. , & Macy, R. J. (2021). The validity and reliability of available intimate partner homicide and reassault risk assessment tools: A systematic review. Trauma, Violence, & Abuse, 22(1), 18–40. 10.1177/1524838018821952 30669956

[jomf12881-bib-0020] Grundy, E. M. , & Tomassini, C. (2010). Marital history, health and mortality among older men and women in England and Wales. BMC Public Health, 10, 554. 10.1186/1471-2458-10-554 20843303 PMC2954998

[jomf12881-bib-0021] Heiskanen, M. , & Ruuskanen, E. (2010). Tuhansien iskujen maa. Miesten kokema väkivalta Suomessa (land of a thousand attacks. Violence experienced by men in Finland). Yhdistyneiden Kansakuntien yhteydessä toimiva Euroopan Kriminaalipolitiikan Instituutti HEUNI.

[jomf12881-bib-0022] Hilton, N. Z. , Harris, G. T. , Rice, M. E. , Lang, C. , Cormier, C. A. , & Lines, K. J. (2004). A brief actuarial assessment for the prediction of wife assault recidivism: The Ontario domestic assault risk assessment. Psychological Assessment, 16(3), 267–275. 10.1037/1040-3590.16.3.267 15456382

[jomf12881-bib-0023] Holmes, T. H. , & Rahe, R. H. (1967). The social readjustment rating scale. Journal of Psychosomatic Research, 11(2), 213–218. 10.1016/0022-3999(67)90010-4 6059863

[jomf12881-bib-0024] Hotton, T. (2001). Spousal violence after marital separation. Canadian Centre for Justice Statistics.

[jomf12881-bib-0025] Idstad, M. , Torvik, F. A. , Borren, I. , Rognmo, K. , Røysamb, E. , & Tambs, K. (2015). Mental distress predicts divorce over 16 years: The HUNT study. BMC Public Health, 15, 320. 10.1186/s12889-015-1662-0 25880080 PMC4394420

[jomf12881-bib-0026] Jalovaara, M. (2013). Socioeconomic resources and the dissolution of cohabitations and marriages. European Journal of Population, 29, 167–193. 10.1007/s10680-012-9280-3

[jomf12881-bib-0027] Jalovaara, M. , & Kulu, H. (2018). Separation risk over union duration: An immediate itch? European Sociological Review, 34(5), 486–500.

[jomf12881-bib-0028] Johnson, H. , & Hotton, T. (2003). Losing control: Homicide risk in estranged and intact intimate relationships. Homicide Studies, 7(1), 58–84. 10.1177/1088767902239243

[jomf12881-bib-0029] Johnson, M. P. (2008). A typology of domestic violence: Intimate terrorism, violent resistance, and situational couple violence. Northeastern University.

[jomf12881-bib-0030] Joung, I. M. A. , Stronks, K. , van de Mheen, H. , van Poppel, F. W. A. , van der Meer, J. B. W. , & Mackenbach, J. P. (1997). The contribution of intermediary factors to marital status differences in self‐reported health. Journal of Marriage and Family, 59(2), 476–490. 10.2307/353484

[jomf12881-bib-0031] Kalmijn, M. (2017). The ambiguous link between marriage and health: A dynamic reanalysis of loss and gain effects. Social Forces, 95(4), 1607–1636. 10.1093/sf/sox015

[jomf12881-bib-0032] Kershner, M. , Long, D. , & Anderson, J. E. (1998). Abuse against women in rural Minnesota. Public Health Nursing, 15(6), 422–431. 10.1111/j.1525-1446.1998.tb00369.x 9874924

[jomf12881-bib-0033] Kingston‐Riechers, J. (2001). The association between the frequency of wife assault and marital dissolution in Canada. Journal of Population Economics, 14(2), 351–365. 10.1007/s001480000055

[jomf12881-bib-0034] Kivelä, S. , Leppäkoski, T. , Ruohoniemi, J. , Puolijoki, H. , & Paavilainen, E. (2019). The documentation and characteristics of hospitalized IPV patients using electronic medical records data: A follow‐up descriptive study. Journal of Family Violence, 34(7), 611–619. 10.1007/s10896-019-00081-z

[jomf12881-bib-0035] Kivivuori, J. , & Lehti, M. (2011). Homicide in Finland and Sweden. Crime and Justice, 40(1), 109–198.

[jomf12881-bib-0036] Kontio, R. (2002). Pahoinpitelyn aiheuttamat kasvovammat (facial bone fractures caused by assaults). Suomen Lääkärilehti (The Finnish Journal of Medicine), 23(57), 2521.

[jomf12881-bib-0037] Le, B. T. , Dierks, E. J. , Ueeck, B. A. , Homer, L. D. , & Potter, B. F. (2001). Maxillofacial injuries associated with domestic violence. Journal of Oral and Maxillofacial Surgery, 59(11), 1277–1283. 10.1053/joms.2001.27490 11688025

[jomf12881-bib-0038] Lee, S. , Cho, E. , Grodstein, F. , Kawachi, I. , Hu, F. B. , & Colditz, G. A. (2005). Effects of marital transitions on changes in dietary and other health behaviours in US women. International Journal of Epidemiology, 34(1), 69–78. 10.1093/ije/dyh258 15231759

[jomf12881-bib-0039] Lehti, M. (2012). Henkirikoskatsaus 2011 (Homicide Report 2011). Oikeuspoliittinen tutkimuslaitos. https://helda.helsinki.fi/handle/10138/152579

[jomf12881-bib-0040] Lehti, M. (2015). Henkirikoskatsaus 2015 (Homicide Report 2015). Kriminologian ja oikeuspolitiikan instituutti. https://helda.helsinki.fi/handle/10138/154148

[jomf12881-bib-0041] Lillard, L. A. , & Waite, L. J. (1995). 'Til death do us part: Marital disruption and mortality. American Journal of Sociology, 100(5), 1131–1156. 10.1086/230634

[jomf12881-bib-0042] Lipsitz, S. R. , Kim, K. , & Zhao, L. (1994). Analysis of repeated categorical data using generalized estimating equations. Statistics in Medicine, 13(11), 1149–1163. 10.1002/sim.4780131106 8091041

[jomf12881-bib-0043] Lövestad, S. , & Krantz, G. (2012). Men's and women's exposure and perpetration of partner violence: An epidemiological study from Sweden. BMC Public Health, 12(1), 945. 10.1186/1471-2458-12-945 23116238 PMC3534228

[jomf12881-bib-0044] Mäenpää, E. (2015). Socio‐economic homogamy and its effects on the stability of cohabiting unions (Finnish yearbook of population research L 2015 supplement). The Population Research Institute.

[jomf12881-bib-0045] Meadows, S. O. , McLanahan, S. S. , & Brooks‐Gunn, J. (2008). Stability and change in family structure and maternal health trajectories. American Sociological Review, 73(2), 314–334. 10.1177/000312240807300207 20333277 PMC2843941

[jomf12881-bib-0046] Messing, J. T. , & Thaller, J. (2015). Intimate partner violence risk assessment: A primer for social workers. The British Journal of Social Work, 45(6), 1804–1820. 10.1093/bjsw/bcu012

[jomf12881-bib-0047] Metsä‐Simola, N. , & Martikainen, P. (2013). Divorce and changes in the prevalence of psychotropic medication use: A register‐based longitudinal study among middle‐aged Finns. Social Science & Medicine, 94, 71–80. 10.1016/j.socscimed.2013.06.027 23931947

[jomf12881-bib-0048] Metsä‐Simola, N. , Martikainen, P. , & Monden, C. W. (2018). Psychiatric morbidity and subsequent divorce: A couple‐level register‐based study in Finland. Social Psychiatry and Psychiatric Epidemiology, 53(8), 823–831. 10.1007/s00127-018-1521-2 29721590

[jomf12881-bib-0049] Ministry of Justice . (2002). Unofficial translation. Marriage act (234/1929; amendments up to 1226/2001 included). Ministry of Justice.

[jomf12881-bib-0050] Ministry of Justice . (2015). Criminal code of Finland. Legally binding only in Finnish and Swedish. Ministry of Justice.

[jomf12881-bib-0051] National Institute for Health and Welfare . (2021). Lähisuhdeväkivalta 2019: Viranomaisten tietoon tulleen lähisuhdeväkivallan määrä kasvussa (Domestic violence 2019: Domestic violence reported to the authorities is increasing). Statistical report 3/2021.

[jomf12881-bib-0052] Nielsen, S. K. , Hardesty, J. L. , & Raffaelli, M. (2016). Exploring variations within situational couple violence and comparisons with coercive controlling violence and no violence/no control. Violence Against Women, 22(2), 206–224. 10.1177/1077801215599842 26333282

[jomf12881-bib-0053] Noone, P. A. (2017). The Holmes–Rahe stress inventory. Occupational Medicine, 67(7), 581–582. 10.1093/occmed/kqx099 29048597

[jomf12881-bib-0054] Office for National Statistics . (2019). The nature of violent crime in England and Wales. A summary of violent crime from the year ending march 2018 crime survey for England and Wales and police recorded crime. Office for National Statistics.

[jomf12881-bib-0055] Pico‐Alfonso, M. A. (2005). Psychological intimate partner violence: The major predictor of posttraumatic stress disorder in abused women. Neuroscience and Biobehavioral Reviews, 29(1), 181–193. 15652265 10.1016/j.neubiorev.2004.08.010

[jomf12881-bib-0056] Pienta, A. M. , Hayward, M. D. , & Jenkins, K. (2000). Health consequences of marriage for the retirement years. Journal of Family Issues, 21(5), 559–586. 10.1177/019251300021005003

[jomf12881-bib-0057] Rahe, R. H. , Veach, T. L. , Tolles, R. L. , & Murakami, K. (2000). The stress and coping inventory: An educational and research instrument. Stress Medicine, 16(4), 199–208. 10.1002/1099-1700(200007)16:4<199::AID-SMI848>3.0.CO;2-D

[jomf12881-bib-0058] Romans, S. , Forte, T. , Cohen, M. M. , Du Mont, J. , & Hyman, I. (2007). Who is most at risk for intimate partner violence? A Canadian population‐based study. Journal of Interpersonal Violence, 22(12), 1495–1514. 10.1177/0886260507306566 17993638

[jomf12881-bib-0059] Rosenfeld, M. J. (2018). Who wants the breakup? Gender and breakup in heterosexual couples. In D. F. Alwin , D. Felmlee , & D. Kreager (Eds.), Social networks and the life course (pp. 221–243). Springer.

[jomf12881-bib-0060] Storey, J. E. , Kropp, P. R. , Hart, S. D. , Belfrage, H. , & Strand, S. (2014). Assessment and management of risk for intimate partner violence by police officers using the brief spousal assault form for the evaluation of risk. Criminal Justice and Behavior, 41(2), 256–271. 10.1177/0093854813503960

[jomf12881-bib-0061] Strohschein, L. , McDonough, P. , Monette, G. , & Shao, Q. (2005). Marital transitions and mental health: Are there gender differences in the short‐term effects of marital status change? Social Science & Medicine, 61(11), 2293–2303. 10.1016/j.socscimed.2005.07.020 16099576

[jomf12881-bib-0062] Tanskanen, M. (2020). Parisuhdeväkivalta (Intimate Partner Violence). In Rikollisuustilanne 2019: Rikollisuuskehitys tilastojen ja tutkimusten valossa (Crime situation 2019: Crime statistics and research). Report nro 42/2020. Institute of Criminology and Legal Policy.

[jomf12881-bib-0063] Turner, E. , Medina, J. , & Brown, G. (2019). Dashing hopes? The predictive accuracy of domestic abuse risk assessment by police. The British Journal of Criminology, 59(5), 1013–1034. 10.1093/bjc/azy074

[jomf12881-bib-0064] Twisk, J. (2003). Applied longitudinal data analysis for epidemiology. A practical guide. Cambridge University Press.

[jomf12881-bib-0065] Umberson, D. (1992). Gender, marital status and the social control of health behavior. Social Science & Medicine (1982), 34(8), 907–917. 10.1016/0277-9536(92)90259-s 1604380

[jomf12881-bib-0066] United Nations Human Rights Committee . (2011). Consideration of reports submitted by states parties under article 40 of the covenant: Sixth periodic reports of states parties: Finland [CCPR/C/FIN/6]. United Nations. Human Rights Committee.

[jomf12881-bib-0067] United Nations Human Rights Committee . (2013). Concluding observations on the sixth periodic report of Finland [CCPR/C/FIN/CO/6]. United Nations. Human Rights Committee.

[jomf12881-bib-0068] Venalainen, S. (2017). “I no longer let anyone hit me for free”: Affective identificatory practices of women imprisoned for violent crimes. NORA ‐ Nordic Journal of Feminist and Gender Research, 25(2), 126–140. 10.1080/08038740.2017.1343256

[jomf12881-bib-0069] Vest, J. R. , Catlin, T. K. , Chen, J. J. , & Brownson, R. C. (2002). Multistate analysis of factors associated with intimate partner violence. American Journal of Preventive Medicine, 22(3), 156–164. 10.1016/S0749-3797(01)00431-7 11897459

[jomf12881-bib-0070] Wade, T. J. , & Pevalin, D. J. (2004). Marital transitions and mental health. Journal of Health and Social Behavior, 45(2), 155–170. 10.1177/002214650404500203 15305757

[jomf12881-bib-0071] Williams, K. , & Dunne‐Bryant, A. (2006). Divorce and adult psychological well‐being: Clarifying the role of gender and child age. Journal of Marriage and Family, 68(5), 1178–1196. 10.1111/j.1741-3737.2006.00322.x

