# Supplementary material for: Partner violence surrounding divorce: A record‐linkage study of wives and their husbands
Source: J Marriage Fam. 2022 Sep 6;85(1):33–54. doi: 10.1111/jomf.12881 (PMC10087196; doi:10.1111/jomf.12881)
Supplement: Supplementary file 1 — Table S1 Trajectories of crime victimization for partner assault and menace before and after divorce for divorcing women without children, divorcing women with the youngest child aged under 12 years, and divorcing women with the youngest child aged 12 years and over, adjusted predicted probabilities, difference in probabilities, and difference in changes in probabilities, Finland. Figure S1. Register‐based study profile describing the selection of women in the hospitalization datasets of divorced (N = 41,766) and continuously married women (N = 304,026) and in the crime datasets of divorced (N = 22,468) and continuously married women (N = 333,542) Figure S2. Trajectories of hospitalization for assault injuries (6‐month probability) before and after the date of divorce for divorcing women, and before and after a random date for continuously married women. The outcome is “corrected” to zero for those whose husband is never registered as a perpetrator against this wife by the police from 2009 to 2017. Figure S3. Trajectories of hospitalization for assault injuries (6‐month probability) before and after the date of separation (the end of cohabitation) for divorcing women, and before and after a random date for continuously married women. [file JOMF-85-33-s001.pdf]

Online Only

Table S1. Trajectories of crime victimization for partner assault and menace before and after divorce for divorcing women without children, divorcing women with the youngest child aged under 12 years, and divorcing women with the youngest child aged 12 years and over, adjusted predicted probabilities, difference in probabilities, and difference in changes in probabilities, Finland.

	No children (N=9,132)		Youngest child aged under 12 years (N=9,770)		Youngest child aged 12 years and over (N=3,566)		Youngest child aged under 12 years	Youngest child aged 12 years and over	Youngest child aged under 12 years	Youngest child aged 12 years and over
Time before/after the year of divorce (years)	Predicted probability (%)	95% CI	Predicted probability (%)	95% CI	Predicted probability (%)	95% CI	Difference in predicted probability between groups (a)	Difference in predicted probability between groups (a)	Difference in changes (b) in predicted probability between groups (a)	Difference in changes (b) in predicted probability between groups (a)
Crime victimization for partner assault										
-3	0.89	[0.70 - 1.08]	0.85	[0.67 - 1.04]	0.60	[0.30 - 0.90]	-0.04	-0.29		
-2	0.91	[0.72 - 1.09]	0.89	[0.70 - 1.07]	0.48	[0.21 - 0.74]	-0.02	-0.43**	0.02	-0.14
-1	1.46	[1.21 - 1.71]	1.79	[1.52 - 2.07]	1.33	[0.88 - 1.78]	0.33*	-0.13	0.37+	0.16
0	0.51	[0.38 - 0.64]	0.99	[0.80 - 1.17]	0.70	[0.39 - 1.02]	0.48**	0.20	0.52**	0.49*
+1	0.14	[0.08 - 0.21]	0.25	[0.16 - 0.33]	0.31	[0.10 - 0.52]	0.10+	0.17	0.14	0.45*
+2	0.10	[0.05 - 0.16]	0.19	[0.12 - 0.27]	0.20	[0.04 - 0.37]	0.09+	0.10	0.13	0.39*
Crime victimization for menace										
-3	0.21	[0.12 - 0.30]	0.19	[0.11 - 0.27]	0.11	[-0.02 - 0.24]	-0.02	-0.10		
-2	0.22	[0.13 - 0.31]	0.22	[0.13 - 0.31]	0.23	[0.04 - 0.41]	0.00	0.00	0.02	0.10
-1	0.46	[0.32 - 0.60]	0.82	[0.63 - 1.01]	0.60	[0.29 - 0.90]	0.36**	0.14	0.38**	0.23
0	0.31	[0.20 - 0.42]	0.71	[0.54 - 0.88]	0.78	[0.43 - 1.13]	0.41**	0.47*	0.43**	0.57**
+1	0.06	[0.02 - 0.11]	0.28	[0.18 - 0.38]	0.22	[0.04 - 0.40]	0.22**	0.16+	0.24**	0.26*
+2	0.08	[0.03 - 0.13]	0.14	[0.07 - 0.20]	0.15	[0.00 - 0.29]	0.06	0.07	0.08	0.17

** p<0.01, * p<0.05, + p<0.10

Note: All predictors at their mean value for probability.

(a) Difference relative to women with no children

(b) Changes relative to the period observed three years prior to the year of divorce

Figure S1. Register-based study profile describing the selection of women in the hospitalization datasets of divorced (N=41,766) and continuously married women (N=304,026) and in the crime datasets of divorced (N=22,468) and continuously married women (N=333,542)

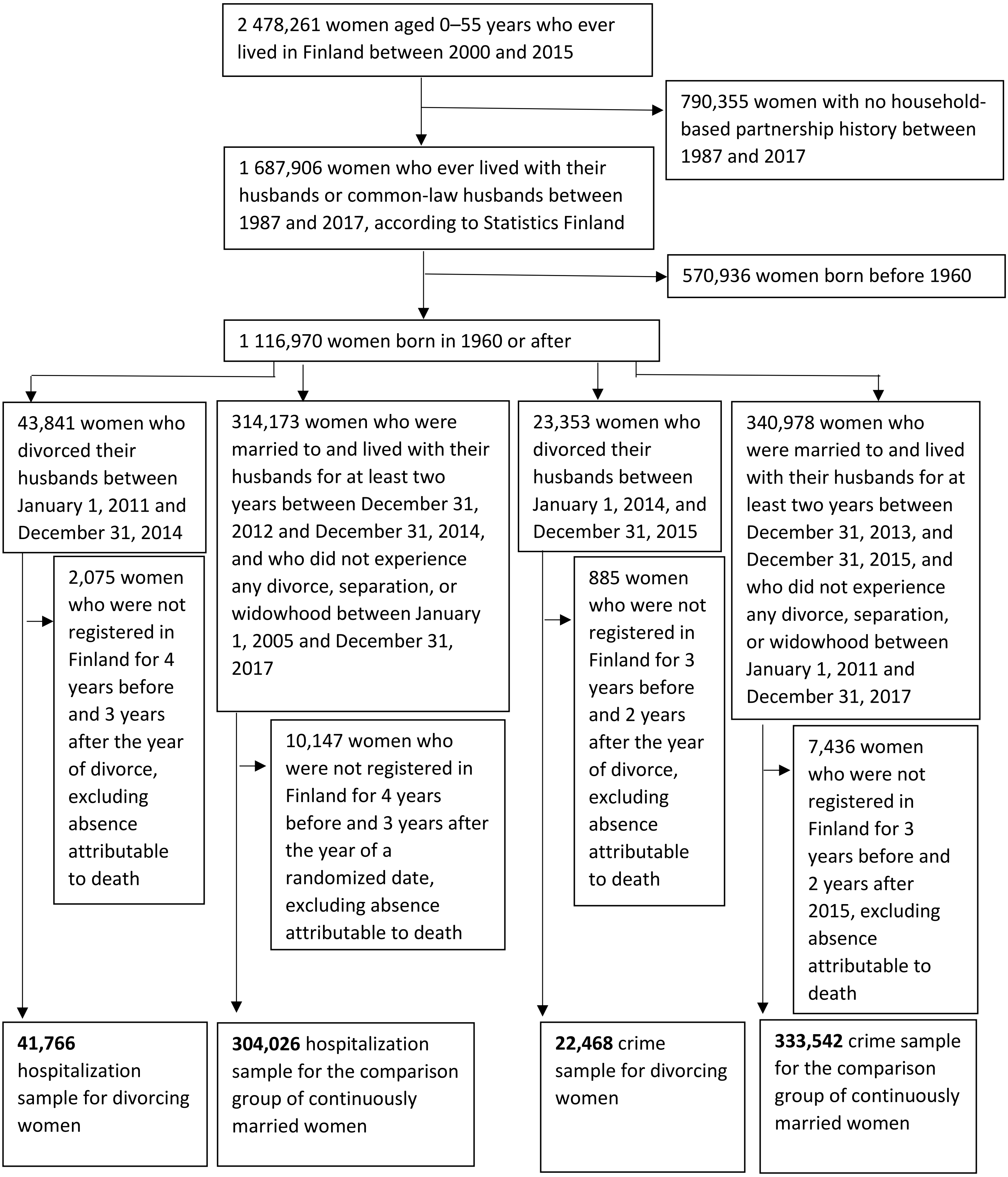

Figure S2. Trajectories of hospitalization for assault injuries (6-month probability) before and after the date of divorce for divorcing women, and before and after a random date for continuously married women. The outcome is “corrected” to zero for those whose husband is never registered as a perpetrator against this wife by the police from 2009 to 2017.

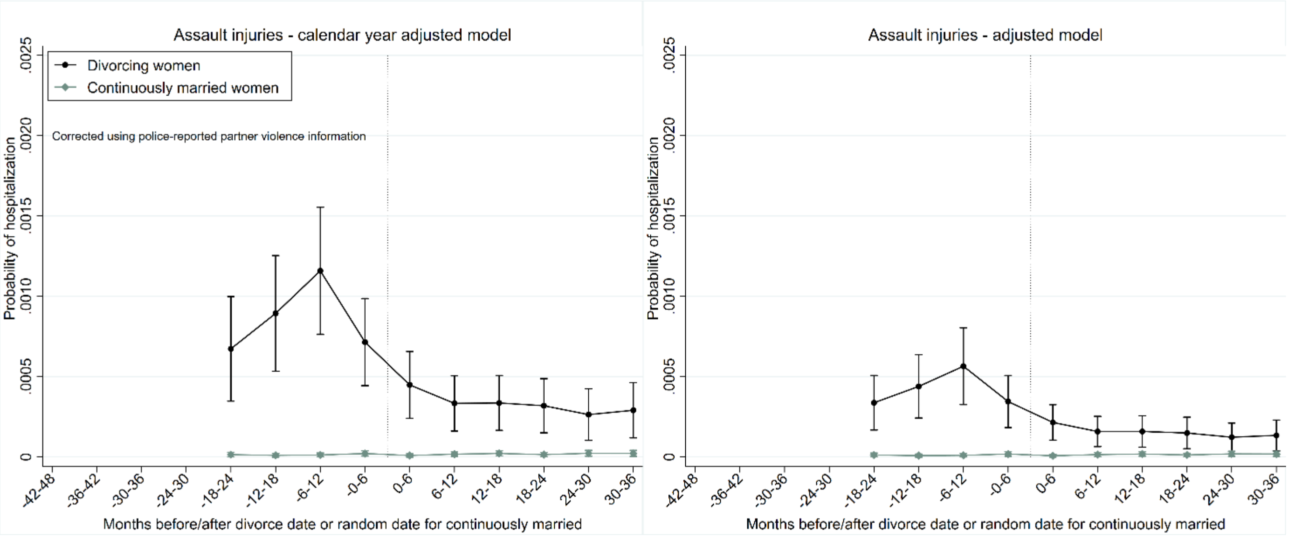

Note: The predicted probabilities shown in the left panel are adjusted for calendar year only and those in the right panel are adjusted for calendar year, age, education, the duration and order of marriage, and the number of children. The results are left truncated due to crime data limitations before 2009.

Figure S3. Trajectories of hospitalization for assault injuries (6-month probability) before and after the date of separation (the end of cohabitation) for divorcing women, and before and after a random date for continuously married women.

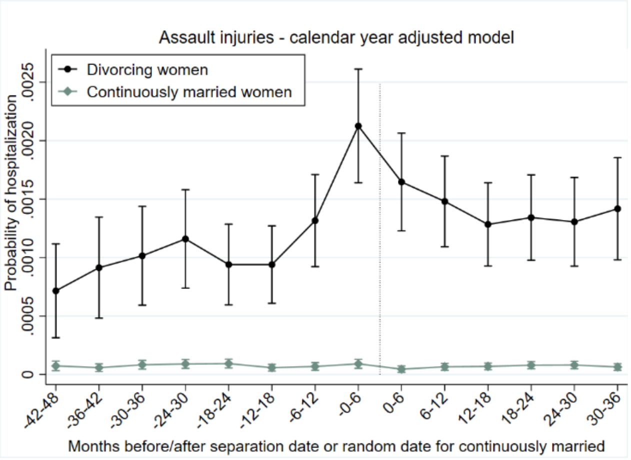

Note: Separations are collected from 2011 to 2014 for women who divorce.
